# Chemokine Receptor CXCR7 Is a Functional Receptor for CXCL12 in Brain Endothelial Cells

**DOI:** 10.1371/journal.pone.0103938

**Published:** 2014-08-01

**Authors:** Yang Liu, Eleanor Carson-Walter, Kevin A. Walter

**Affiliations:** 1 Department of Neurosurgery, University of Rochester School of Medicine and Dentistry, Rochester, New York, United States of America; 2 Wilmot Cancer Center, University of Rochester School of Medicine and Dentistry, Rochester, New York, United States of America; UAE University, Faculty of Medicine & Health Sciences, United Arab Emirates

## Abstract

The chemokine CXCL12 regulates multiple cell functions through its receptor, CXCR4. However, recent studies have shown that CXCL12 also binds a second receptor, CXCR7, to potentiate signal transduction and cell activity. In contrast to CXCL12/CXCR4, few studies have focused on the role of CXCR7 in vascular biology and its role in human brain microvascular endothelial cells (HBMECs) remains unclear. In this report, we used complementary methods, including immunocytofluorescence, Western blot, and flow cytometry analyses, to demonstrate that CXCR7 was expressed on HBMECs. We then employed short hairpin RNA (shRNA) technology to knockdown CXCR7 in HBMECs. Knockdown of CXCR7 in HBMECs resulted in significantly reduced HBMEC proliferation, tube formation, and migration, as well as adhesion to matrigel and tumor cells. Blocking CXCR7 with a specific antibody or small molecule antagonist similarly disrupted HBMEC binding to matrigel or tumor cells. We found that tumor necrosis factor (TNF)-α induced CXCR7 in a time and dose-response manner and that this increase preceded an increase in vascular cell adhesion molecule-1 (VCAM-1). Knockdown of CXCR7 resulted in suppression of VCAM-1, suggesting that the reduced binding of CXCR7-knockdown HBMECs may result from suppression of VCAM-1. Collectively, CXCR7 acted as a functional receptor for CXCL12 in brain endothelial cells. Targeting CXCR7 in tumor vasculature may provide novel opportunities for improving brain tumor therapy.

## Introduction

Chemokine CXCL12 (stromal cell derived factor 1α or SDF-1α) is a key player in angiogenesis and impacts such diverse processes as inflammation, wound healing, embryonic development, and the growth of malignant tumors. CXCL12 promotes tubulogenesis and migration of endothelial cells, including human umbilical endothelial cells (HUVECs) and human microvascular endothelial cells (HMVECs) [1]–[3], largely through binding chemokine receptor 4 (CXCR4) [4]–[7].

Recently, CXCR7 was recognized to be a second receptor for CXCL12 [8], [9]. In contrast to the extensive studies regarding CXCR4, fewer studies have investigated the role of CXCR7 in endothelial biology. CXCR7 is expressed by human endothelial cells, including HUVECs [9]–[12], HMVECs [13], pulmonary microvascular endothelial cells [14], and endothelial cells within the central nervous system [15]. CXCR7−/− mice die in the first week after birth due to abnormal cardiovascular development, suggesting a critical role for CXCR7 in endothelial biology and cardiac development [16], [17]. We originally identified CXCR7 (then called RDC1) as a selective marker of glioblastoma (GBM) derived microvascular endothelial cells and confirmed that it was induced in the tumor endothelium of both primary and metastatic malignant brain tumors [18], [19]. CXCR7 expression increased with brain tumor grade [20], [21] and elevated CXCR7 mRNA levels correlate with poor survival in glioma patients (Liu Y, unpublished data). Aberrant CXCR7 expression has subsequently been reported in the vasculature of a variety of cancer models [20]–[26], indicating that CXCR7 may actively contribute to tumor angiogenesis and/or vasculogenesis. However, the function of CXCR7 in brain endothelium has remained unclear. In this study, we investigated the role of CXCL12/CXCR7 signaling in human brain microvascular endothelial cells (HBMECs). We found that CXCR7 regulates multiple HBMEC functions including proliferation, tube formation, migration, adhesion, and binding to GBM cells. We further demonstrated that CXCR7 in HBMECs can be up-regulated by tumor necrosis factor (TNF)*-*α and is an upstream gene of VCAM-1.

## Materials and Methods

### Cells and Reagents

Primary human brain microvascular endothelial cells (HBMECs) were from Cell Systems (Kirkland, WA) and maintained in complete medium with serum and CultureBoost (Cell Systems). All primary HBMECs cultures were used between passage 4 and 9. GBM tumor cells U251MG cells and U373MG cells were gifts of Dr. John Laterra (Johns Hopkins School of Medicine) and originally purchased from the American Type Culture Collection (ATCC). U251MG cells were maintained as previously described [27]. U373MG cells were maintained in DMEM (Invitrogen) with 10% FBS, 20mM HEPES (Invitrogen) and 1% penicillin-streptomycin. Primary CXCR7 antibody (11G8) and mouse IgG1 isotype control for immunocytofluorescence and neutralization experiments were from R&D Systems (catalogue numbers: MAB42273 and MAB002, respectively). Primary CXCR7 and VCAM-1 antibodies for immunoblot analysis were from GeneTex (catalogue numbers: GTX100027 and GTX110684, respectively). Human CXCR7 phycoerythrin antibody and isotype control for flow analysis were from R&D Systems (catalogue numbers: FAB42271P and IC003P, respectively). Recombinant human CXCL12/SDF-1α and TNF-α were from Peprotech (Rocky Hill, NJ).

### Immunocytofluorescence

HBMECs were plated on culture slides (BD, catalogue number: 354118) at a density of 6×10^4^/well on day 1. On the second day, cells were washed with PBS, followed by fixation with 3.2% paraformaldehyde (PFA) at room temperature for 15 min. Cells were blocked with 10% normal donkey serum for 1 hour and incubated with primary antibody (11G8, 1∶200) for 1 hour. Cells were washed with PBS and incubated with donkey anti-mouse Alexa Fluor 594 (1∶1000; Invitrogen, catalogue number: A21203) for 1 hour, washed and mounted with Prolong Gold antifade reagent with DAPI (Invitrogen). Images were acquired on an Olympus IX71 inverted microscope with DP controller (3.2) software.

### Construction of shRNA-CXCR7 HBMEC

GIPZ lentiviral shRNAmir plasmids expressing short hairpin RNA (shRNA) targeted to CXCR7 and scrambled control sequences were purchased from Thermo Scientific (Waltham, MA). Recombinant lentiviruses were produced by co-transfecting human embryonic kidney 293T cells with the GIPZ lentiviral vectors and translenti viral GIPZ packaging system (Thermo Scientific) using X-tremeGENE HP DNA transfection reagent (Roche Diagnostics, Indianapolis, IN) according to manufacturer’s protocol. HBMECs were infected with lentiviruses at 0.5 multiplicity of infection, followed by selection with puromycin (0.5 ug/ml). Stably expressing shRNA-scramble and shRNA-CXCR7 lines were designated scramble-HBMECs (SC) and p148-HBMECs (p148), respectively. Lack of sequence homology between CXCR7, CXCR4, VCAM-1 and RefSeq genomes was confirmed using BLAST (NIH) to ensure that all shRNA-CXCR7 constructs were specifically targeted to CXCR7.

### RNA isolation and reverse transcription

Total RNA was isolated using Qiagen RNeasy Mini kit (Qiagen, Qiagen Sciences, MD) according to manufacturer’s protocol. cDNA was generated using iScript cDNA Synthesis kit (Bio-Rad, Hercules, CA) according to manufacturer’s protocol.

### Quantitative real-time PCR (qPCR)

qPCR was performed by using iQ5 real-time PCR detection system (Bio-Rad) as previously described [19]. CXCR7 and GAPDH primers were previously reported [19]. CXCR4 forward primer: CCTCCTGCTGACTATTCCCGA, reverse primer: GGAACACAACCACCCACAAGT. Vascular cell adhesion molecule-1 (VCAM-1) primers were described in a previous publication [28]. All primers were synthesized by Integrated DNA Technologies (IDT, Coralville, IA). Samples were analyzed in triplicates.

### Western blotting

Total protein was extracted from shRNA-CXCR7 or scramble HBMECs using RIPA buffer (Thermo Scientific) and quantified using Bio-Rad DC protein assay kit (Bio-Rad). Thirty micrograms of total protein was loaded on a Novex 10% Tris-Glycine gel (Life Technologies), electrophoresed and transferred to a PVDF membrane. Blots were incubated with CXCR7 or VCAM-1 primary antibody (1∶250) at 4°C overnight, followed by goat anti-rabbit horseradish peroxidase secondary antibody (1∶2000) for 1 hour at room temperature and visualized with Immobilon Western Chemiluminescent HRP Substrate (Millipore). To control protein loading, blots were probed with goat anti-actin (1∶1000, Santa Cruz) or mouse anti-GAPDH (1∶5000, Abcam) and 1∶10,000 secondary antibody.

### Flow cytometry analysis

To examine cell surface expression of CXCR7, shRNA-CXCR7 or scramble HBMECs were collected and immediately washed with ice-cold Miltenyi Washing Buffer (MWB), then blocked with diluted human FcR blocking reagent (MACS Miltenyi Biotec) for 15 min at room temperature. Cells were further incubated with human CXCR7-PE or isotype IgG2A-PE for 30 minutes on ice, followed by washing with cold MWB. Samples were read on a FACSAria IIIU (Becton Dickinson, San Jose, CA) and analyzed with FlowJo cytometry analysis software. Ten thousand events were collected per sample.

### Cell proliferation assay

Cell growth was measured using the Cell Proliferation Kit II (Roche Applied Science, Mannheim, Germany) according to manufacturer’s protocol. Briefly, shRNA-CXCR7 or scramble HBMECs were seeded at 1000 cells/well in 96-well flat bottom plate (BD Bioscience) in the presence of 200 ng/ml CXCL12. At the indicated time, 50 µl XTT labeling mixture was added and cells were incubated at 37°C for 4 hours followed by measuring absorbance at 450 nm with a reference wavelength at 620 nm on SpectraMax M2 microplate reader (Molecular Devices).

### Tubulogenesis assay

Tubulogenesis assays were performed as described [27]. Briefly, shRNA-CXCR7 or scramble HMBECs were suspended in 1∶10 diluted complete medium (Cell Systems) supplemented with 200 ng/ml CXCL12. Cells were seeded at 6×10^4^ cells/well in 24-well plate coated with 200 µl matrigel (BD bioscience, catalogue number: 356237). Six hours later, five high power fields were randomly photographed for each condition and quantified by ImageJ software (NIH, http://rsbweb.nih.gov/ij).

### Transwell migration assay

Transwell migration assays were performed using BD Biocoat growth factor reduced matrigel invasion chambers (24 well plate, 8 micron) according to the manufacturer’s protocol (BD Bioscience). Briefly, invasion chambers were prehydrated with 500 µl/well serum-free medium (Cell Systems) for 2 hours at 37°C in 5% CO_2_. 2.5×10^4^ shRNA-CXCR7 or scramble HBMECs were suspended in 500 µl 10% complete medium (Cell Systems) and seeded on the upper chamber while the lower chamber was filled with 600 µl 10% complete medium with CXCL12 (200 ng/ml). Cells were allowed to migrate for 24 hours at 37°C in a 5% CO_2_ incubator. After 24 hours, cells remaining on the upper membrane surface were removed with a Q-tip and cells remaining on the bottom side of membrane were stained with 10 µM cell tracker (Invitrogen). Five high power fields of adherent cells were counted randomly in each well under an inverted microscope and averaged for statistical analysis.

### Adhesion assays

HBMEC adhesion assays were performed as previously described with minor modifications [29]. Briefly, GFP expressing shRNA-CXCR7 or scramble HBMECs were seeded at 8×10^3^/well in 96 well plates coated with 50 µl matrigel (BD Biosciences). After a one hour incubation at 37°C, adherent cells were washed with PBS, fixed with 3.2% PFA, and rinsed with PBS again. Adherent cells were quantified by fluorescence on a SpectraMax M2 microplate reader (Molecular Devices). For intercellular adhesion (cell binding) assays, U251MG and U373MG GBM cells were seeded at a density of 1×10^4^/well in a 96 well plate on day 1. On the second day, tumor cells formed a confluent cell layer and 8×10^3^ shRNA-CXCR7 or shRNA scramble HBMECs were seeded on the top of tumor cells and adhesion was measured as described above.

For CXCR7 neutralization and blocking experiments, U251MG and U373MG GBM cells were seeded at a density of 1×10^4^/well in a 96 well plate on day 1. On day 2, HBMECs were stained with carboxyfluorescein succinimidyl ester (CFSE, Molecular Probes) and incubated with 10 µg/ml of CXCR7 specific antibody (11G8), 10 µg/ml control mouse IgG1, 1 µM of CXCR7 targeted small molecule antagonist CCX771 (kind gift of Dr. Mark Penfold, Chemocentryx, Inc.) or 1 µM control CCX704 (Dr. Penfold) for 30 min. at room temperature. 8×10^3^ of HBMECs were then seeded on the top of the tumor cells or matrigel and adhesion was measured as described above.

### Statistical analysis

Results were analyzed by a two-tailed Student’s t test and differences with *p*<0.05 were considered to be statistically significant. All experiments were repeated at least three times and data are shown as means ± S.D.

## Results

### CXCR7 is expressed by HBMECs

**Figure 1:**
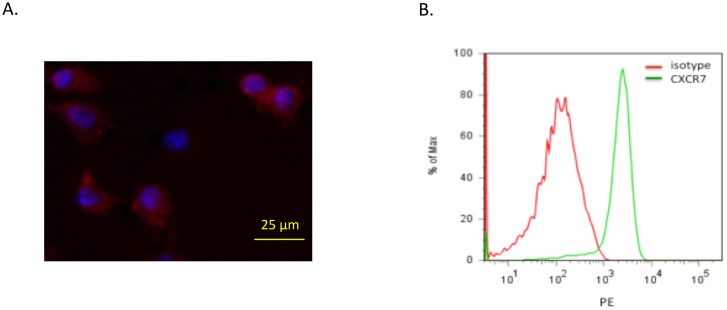
CXCR7 is expressed in HBMECs. *A,* Representative immunocytofluorescence image for CXCR7 (red) in HBMECs. Nuclei are stained with DAPI (blue). *B,* Flow cytometry analysis for CXCR7 in HBMECs. HBMECs were stained with CXCR7 specific primary antibody and subjected to immunocytofluorescence and flow cytometry, respectively, as described in “Materials and Methods”.

In order to study CXCR7 function in brain endothelial cells, we first examined basal expression of CXCR7 in HBMECs by immunocytofluorescence and flow cytometry. As shown in Fig. 1*A*, immunocytofluorescence demonstrated positive cytoplasmic and surface expression of CXCR7 (red) in HBMECs. Flow cytometric analysis of non-permeabilized HBMECs confirmed that CXCR7 was expressed in HBMECs and localized to the cell surface (Fig. 1*B*). We also performed flow cytometry on permeabilized cells and found considerable levels of intracellular CXCR7 in HBMECs (data not shown). We did confirm expression of CXCR4 in HBMECs by qPCR and found that CXCR4 expression was comparable to CXCR7 (data not shown). However, since CXCR4 has been extensively studied [4]–[7], we focused specifically on the role of CXCR7 in HBMECs.

### CXCR7 knockdown attenuates multiple HBMEC functions including proliferation, tube formation, migration, adhesion, and binding to tumor cells

**Figure 2:**
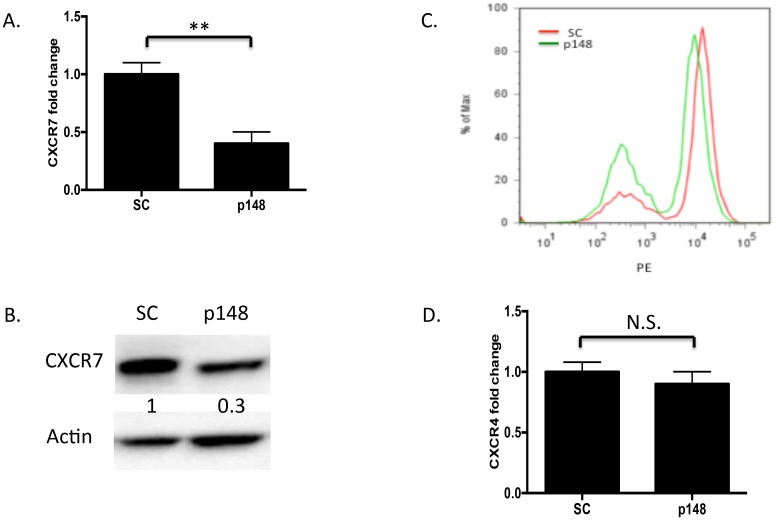
Knockdown of CXCR7 by RNA interference. HBMECs stably infected with CXCR7-targeted shRNA or scrambled control shRNA lentiviruses were subjected to qPCR, Western blot and flow cytometry analysis, respectively, as described in “Materials and Methods”. *A,* Total RNA from shRNA-CXCR7 and shRNA-scramble HBMECs was analyzed for CXCR7 by qPCR. Data are shown as means ± S.D. from three independent experiments. **, *p*<0.01. *B.* Total protein from shRNA-CXCR7 and shRNA-scramble HBMECs was analyzed by Western blot. Three independent experiments were performed and representative data are shown. *C.* shRNA-scramble and shRNA-CXCR7 HBMECs were collected from 6 well plates and subjected to flow cytometry analysis. Three independent experiments were performed and representative data are shown. *D.* Total RNA from shRNA-CXCR7 and shRNA-scramble HBMECs was analyzed for CXCR4 by qPCR. Data are shown as means ± S.D from three independent experiments. *N.S.,* not significant. SC: shRNA-scramble HBMECs; p148: shRNA-CXCR7 HBMECs.

To interrogate the function of CXCR7 in HBMECs, we generated lentiviral constructs expressing shRNA specific to CXCR7 or a nonspecific scrambled control shRNA to suppress CXCR7 in HBMECs. Stably infected HBMECs were selected by puromycin resistance and transgene expression was confirmed by GFP fluorescence. CXCR7 mRNA expression was suppressed over 60% compared to control, scrambled cells (Fig. 2*A*). When the identical lentivirus was used to infect glioma cells, CXCR7 mRNA was suppressed over 90% (data not shown), indicating that the efficacy of the lentiviral knockdown may depend on cell type. Suppression of CXCR7 protein was confirmed by Western blot analysis and flow cytometry (Fig. 2*B* and 2*C*). To rule out potential off-target effects of shRNA-CXCR7 on CXCR4, we performed qPCR to check CXCR4 on scramble HBMECs and p148-HBMECs and demonstrated that CXCR4 expression remained unchanged (Fig. 2*D*).

**Figure 3:**
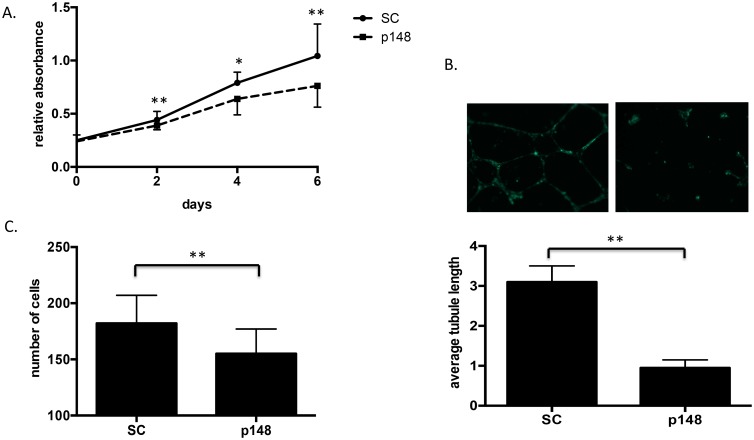
CXCR7 regulates multiple functions in HBMECs. *A.* shRNA-scramble CXCR7 or shRNA-CXCR7 HBMECs were seeded on 96 well plate in the presence of 200 ng/ml CXCL12 and cell proliferation was analyzed by XTT assay as described in “Materials and Methods”. *B.* shRNA-scramble or shRNA-CXCR7 HBMECs were seeded on 24 well plate coated with matrigel and tubulogenesis assay was performed as described in in “Materials and Methods”. *C.* shRNA-scramble CXCR7 or shRNA-CXCR7 HBMECs were seeded on the upper chamber of BD matrigel invasion chambers while the lower chamber was filled with 600 µl 10% complete medium with CXCL12 (200 ng/ml). Cell migration ability was analyzed as described in “Materials and Methods”. Data are shown as means ± S.D from three independent experiments. *, *p*<0.05; **, *p*<0.01; SC: shRNA-scramble HBMECs; p148: shRNA-CXCR7 HBMECs.

We next used the stable CXCR7 knockdown and scrambled control HBMECs (p148-HBMECs and SC-HBMECs) to study cell proliferation, tubulogenesis, migration, and adhesion. We found that compared to SC-HBMECs, p148-HBMECs showed significant reduction of proliferation even in the presence of CXCL12 (Fig. 3*A*). The p148-HBMECs also demonstrated significantly reduced tubulogenesis in the presence of CXCL12, both as measured by tubule number and length (Fig. 3*B*). Additionally, transwell migration of p148-HBMECs in response to CXCL12 was significantly reduced (Fig. 3*C*). Finally, when we used small interfering RNA (siRNA) targeting a non-overlapping sequence of CXCR7 to transiently knockdown CXCR7 in HBMECs, we found that suppression of CXCR7 similarly impeded HBMEC proliferation, tubule formation and migration (data not shown).

**Figure 4:**
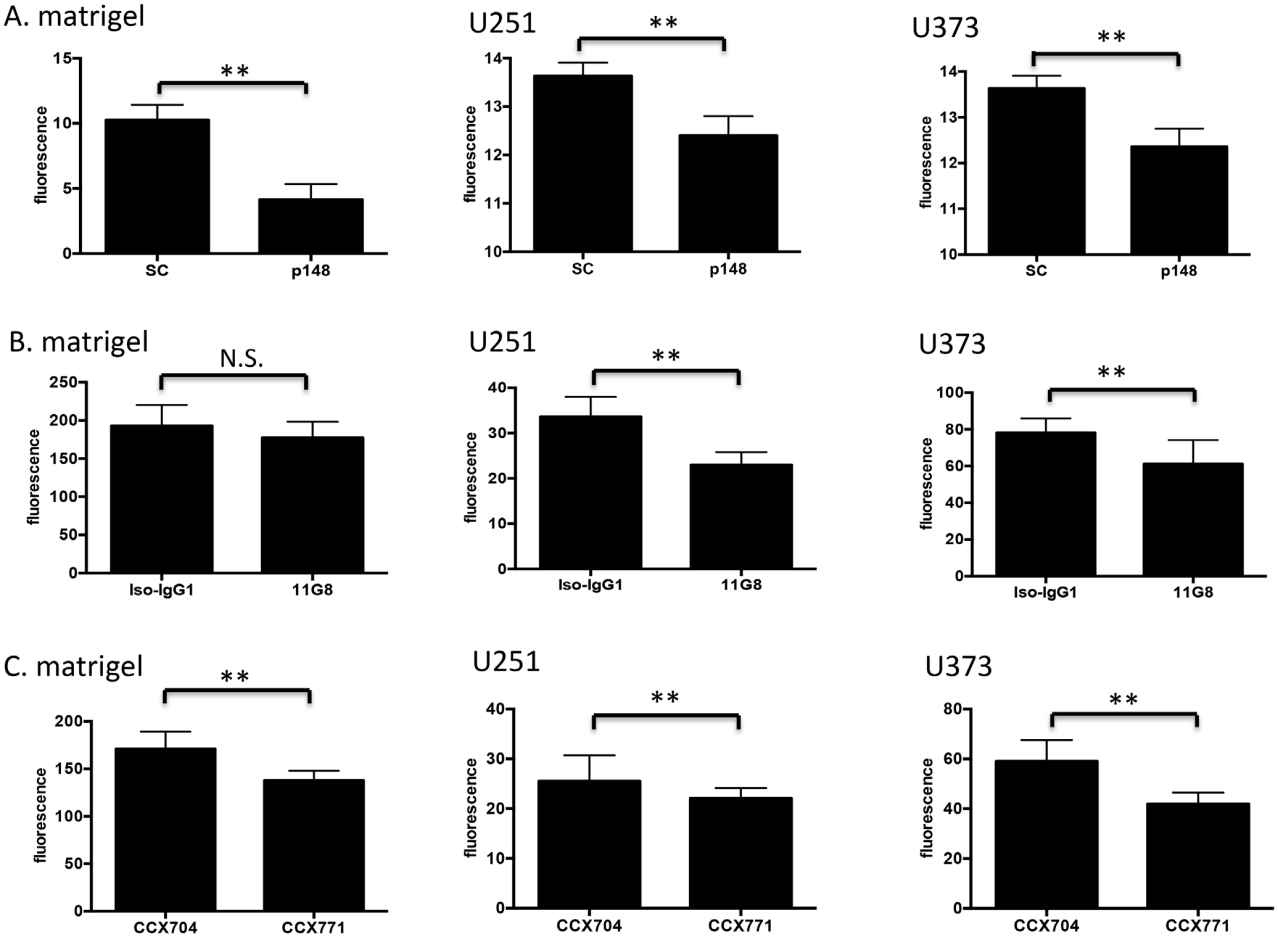
CXCR7 regulates HBMEC adhesion and binding to GBM cells. *A.* shRNA-scramble HBMECs or shRNA-CXCR7 HBMECs were seeded on matrigel or GBM cells (U251 and U373) and adhesion was measured as described in “Materials and Methods”. *B,* HBMECs were pretreated with CXCR7 specific antibody (11G8) or isotype-IgG1 for 30 min. and subsequent adhesion was measured as described in “Materials and Methods”. *C.* HBMECs were pretreated with a CXCR7 small molecule antagonist, CCX771, or negative control, CCX704, for 30 min. and adhesion assays were performed as described in “Materials and Methods”. Data are shown as means ± S.D from three independent experiments. **, *p*<0.01; *N.S.,* not significant; SC: shRNA-scramble HBMECs; p148: shRNA-CXCR7 HBMECs.

Since GBM is a highly invasive tumor and its cells track along existing blood vessels [30], we examined how HBMECs interacted with extracellular matrix and tumor cells after suppression of CXCR7. We found that suppression of CXCR7 significantly disrupted HBMEC binding to an extracellular matrix as well as GBM cell lines U251 and U373 (Fig. 4*A*). To confirm these findings, we used a CXCR7 specific antibody, 11G8, to neutralize surface CXCR7, as well as a small molecule antagonist, CCX771, to block CXCR7 in HBMECs and measured subsequent cellular adhesion to matrigel and glioma cells. We found that both the blocking antibody and small molecule inhibitor significantly attenuated HBMEC binding to matrigel and tumor cells (Fig. 4B and 4*C*). Collectively, these data indicate that CXCR7 can actively contribute to adhesion between brain endothelial cells and tumor cells.

### CXCR7 can be induced by TNF-α in brain endothelial cells

**Figure 5:**
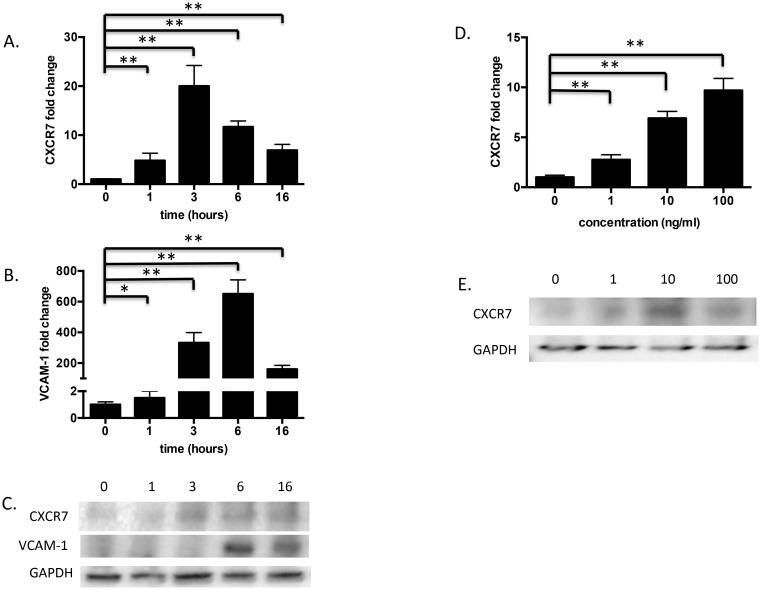
CXCR7 is induced by TNF-α in a time and dose-dependent manner. HBMECs were treated with 10/ml of TNF-α and collected at 0, 1, 3, 6, or 16 hours or treated with increasing concentrations (1, 10, 100 ng/ml) of TNF-α for 24 hours. *A, B,* and *D,* Total RNA from TNF-α-treated HBMECs was analyzed by qPCR as described in “Materials and Methods”. Data are shown as means ± S.D from three independent experiments. *, *p*<0.05; **, *p*<0.01. *C* and *E,* Total protein from TNF-α-treated HBMECs was analyzed by Western blot as described in “Materials and Methods”. Three independent experiments were performed and representative data are shown.

TNF*-*α can induce CXCR7 mRNA in HUVEC cells [9], [31]. Recognizing that an inflammatory microenvironment contributes to GBM progression and that TNF*-*α is a key pro-inflammatory mediator, we asked if TNF-α could regulate CXCR7 in HBMECs. We treated HBMECs with 10 ng/ml of TNF-α for 0, 1, 3, 6, or 16 hours or treated with increasing concentrations (1, 10, 100 ng/ml) of TNF-α for 24 hours. As shown in Fig. 5*A*, TNF*-*α treatment (10 ng/ml) triggered a rapid increase of CXCR7 mRNA in HBMEC cells, beginning as early as 1 hour after treatment and peaking at 3 hours after addition. This was confirmed by Western blot (Fig. 5*C*). Lower doses of TNF*-*α (1 ng/ml) induced CXCR7 to a lesser degree at both the mRNA and protein levels (Fig. 5*D* and 5*E*). Flow cytometry verified that TNF-α induced CXCR7 protein (data not shown). Overall, CXCR7 was up-regulated by TNF*-*α in a time and dose-dependent manner.

### CXCR7 is an upstream gene of VCAM-1

**Figure 6:**
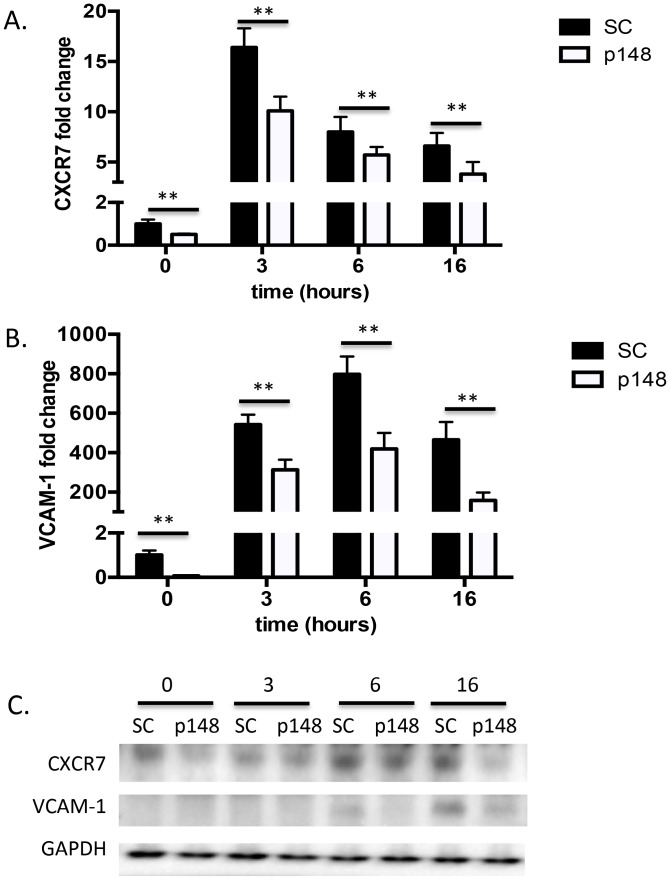
CXCR7 is an upstream gene of VCAM-1 in HBMECs. shRNA-scramble or shRNA-CXCR7 scramble HBMECs were treated with 10 ng/ml TNF-α and collected at indicated time points. *A* and *B*. Total RNA from TNF-α-treated shRNA-scramble or shRNA-CXCR7 HBMECs was analyzed by qPCR as described in “Materials and Methods”. Data are shown as means ± S.D from three independent experiments. **, *p*<0.01. *C,* Total protein from TNF-α-treated shRNA-CXCR7 or scramble HBMECs was analyzed by Western blot as described in “Materials and Methods”. Three independent experiments were performed and representative data are shown. SC: shRNA-scramble HBMECs; p148: shRNA-CXCR7 HBMECs.

VCAM-1 is a key mediator of intercellular contact in tumors and has been suggested as a potential therapeutic target in cancer metastasis [32] and GBM cell invasion [33]. Since CXCR7 knockdown significantly suppressed HBMEC adhesion to matrigel and glioma cell lines, we questioned whether this could be due to a coordinate change in VCAM-1 levels. In response to TNF*-*α treatment, CXCR7 expression increased, peaking at 3 hours, followed by a peak induction of VCAM-1 expression at 6 hours (Fig. 5*A–C*). Subsequently, we asked if CXCR7 could regulate VCAM-1 in HBMECs. We found that suppression of CXCR7 by shRNA blocked the induction of VCAM-1 by TNF*-*α at both mRNA and protein levels (Fig. 6). Collectively, these data suggest that CXCR7 functions upstream of VCAM-1 in HBMECs.

## Discussion

Malignant gliomas are characterized by abundant neovascularization, a hallmark that correlates directly with clinical recurrence and inversely with the post-operative survival. Radiation and chemotherapy can damage tumor vasculature and inhibit tumor angiogenesis. Recovery of the tumor microvasculature is mediated, in part, by CXCL12 and CXCR4 [34], [35], but the role of receptor CXCR7 remains controversial. We and other groups found that CXCR7 was highly expressed in the malignant brain tumor vasculature [18]–[20], [23], increased with brain tumor grade [20], [21] and correlated with poor survival in glioma patients (Liu Y, unpublished data). However, despite the fact that CXCR7 is highly expressed in tumor vasculature, the functional role of CXCR7 in brain endothelial cells remains poorly understood. Although two recent studies showed that CXCR7 expression is sufficient to drive pulmonary and blood vascular endothelial cell growth [14], [36], these results are not necessarily translatable to the cerebrovasculature, which is characterized by unique blood brain barrier biology and gene expression profiles. In this report, we used HBMECs to study the role of CXCR7 in normal brain endothelial cells *in vitro*. We confirmed that CXCR7 was expressed on HBMECs (Fig. 1), consistent with a recent report [37]. Furthermore, we found that suppression of CXCR7 by targeted shRNA significantly inhibited multiple aspects of brain endothelial cell function including proliferation, tube formation, migration and adhesion (Fig. 3–4). To validate these findings, we demonstrated that transient knockdown of CXCR7 by siRNA in HBMECs led to similarly significant reductions in proliferation, tube formation, and migration (data not shown). Since tubulogenesis, unlike proliferation, is measured in hours, the decrease seen in tubule formation was due to altered acute cellular responses to the microenvironment, not long term growth retardation. Furthermore, inhibition of HBMEC responses to CXCL12 was observed despite the continued expression of CXCR4 in these cells. Collectively, these data suggest that CXCR7 actively participates in brain microvascular endothelial cell growth and angiogenesis. We also found that CXCR4 expression level in HBMECs is comparable to CXCR7 and blockage of CXCR4 with its antagonist AMD3100 significantly impaired HBMECs tube formation and transwell migration (data not shown). Thus, selectively targeting both CXCR7 and CXCR4 in tumor vasculature may provide a robust anti-angiogenic approach to GBM treatment.

The CXCL12 pathway is a key receptor system in the cross-talk between tumor cells and the vasculature in glioma [37]–[39]. Increased CXCL12/CXCR4 signaling has been reported after withdrawal of chemotherapy and shown to promote bone marrow derived endothelial progenitor cell mobilization and subsequent tumor homing, leading to an angiogenic rebound and regrowth of tumors [34], [40], [41]. CXCR7 has been suggested to regulate cell adhesion in tumor cells [9], [42], [43], endothelial progenitor cells [44], [45] and renal multipotent progenitors [46]. In the central nervous system, CXCR7 was shown to modulate the adhesion of leukocytes to the microvasculature [15]. Recently, it was proposed that inhibition of CXCR7 on endothelial cells coupled with inhibition of CXCR4 on CD11b+ monocytes can inhibit the post-irradiation recovery of the vasculature and delay local tumor recurrence [35]. In this study we showed that stable knockdown of CXCR7 on HBMECs significantly inhibited brain endothelial cell binding to tumor cells (Fig. 4*A*). Likewise, blocking CXCR7 with a neutralizing antibody (11G8) or small molecule antagonist (CCX771) similarly reduced HBMEC binding ability to matrigel and GBM cells (Fig. 4*B* and 4*C*). This suggests that CXCR7 actively contributes to physical interactions between brain endothelial cells and their environment and may contribute to glioma cell invasion along vascular tracks. Disruption of endothelial-tumor cell adhesion may be a mechanism to prevent GBM invasion as well as recurrence.

TNF-α can directly and indirectly promote GBM tumorigenesis and angiogenesis [47], [48]. TNF-α expression levels can be elevated in GBM endothelial cells [49]. We examined whether TNF-α induced CXCR7 in HBMECs. We found that TNF-α induced CXCR7 expression in HBMECs at both the mRNA and protein level in a time- and dose-dependent manner (Fig. 5), indicating crosstalk between the TNF-α and CXCL12 signaling pathways.

Our results also reveal that CXCR7 can regulate VCAM-1 in HBMECs. CXCR7 has been reported to influence adhesion in multiple cell types and can specifically regulate cadherin 11 and CD44 in prostate cancer cells [42]. We found that suppression of CXCR7 significantly repressed VCAM-1 expression at both mRNA and protein levels with and without TNF-α stimulation (Fig. 6). Thus, CXCR7 may regulate endothelial adhesion, at least in part, through modulation of downstream VCAM-1 levels and targeting CXCR7/VCAM-1 may provide a novel opportunity to prevent tumor invasion and metastasis.

In conclusion, our study demonstrates that CXCR7 can regulate multiple proliferative functions in brain endothelial cells and can, itself, be regulated by TNF-α. CXCR7 mediates endothelial cell adhesion to GBM cells and endothelial expression of the adhesion molecule, VCAM-1. Targeted inhibition of vascular CXCR7 may have direct anti-angiogenic or anti-metastatic benefits for brain tumor patients.
